# Development and Validation of a Quantitative RT-qPCR Panel for the Detection and Monitoring of Polioviruses in Wastewater Samples

**DOI:** 10.3390/microorganisms14030709

**Published:** 2026-03-21

**Authors:** Linnet Immaraj, Judy Y. Qiu, Logan A. Brand, Tiejun Gao, Bonita Lee, Michael Parkins, Casey Hubert, Christine O’Grady, Xiaoli Pang

**Affiliations:** 1 Department of Laboratory Medicine and Pathology, University of Alberta, Edmonton, AB T6G 2J2, Canada; immaraj@ualberta.ca (L.I.); judy.qiu@albertaprecisionlabs.ca (J.Y.Q.); lbrand@ualberta.ca (L.A.B.); tiejun1@ualberta.ca (T.G.); 2 Public Health Laboratory, Alberta Precision Laboratories, Edmonton, AB T6G 2J2, Canada; 3 Department of Pediatrics, University of Alberta, Edmonton, AB T6G 2B7, Canada; bonita.lee@albertahealthservices.ca; 4 Department of Microbiology, Immunology and Infectious Diseases, University of Calgary, Calgary, AB T2N 1N4, Canada; mdparkin@ucalgary.ca; 5 Department of Biological Sciences, University of Calgary, Calgary, AB T2N 1N4, Canada; chubert@ucalgary.ca; 6 ACWA, University of Calgary, Calgary, AB T2N 1N4, Canada; christine.ogrady@ucalgary.ca

**Keywords:** RT-qPCR panel, poliovirus, IPV, wastewater, Sabin, WPV

## Abstract

Clusters of acute flaccid paralysis (AFP) caused by oral vaccine-derived poliovirus (VDPV) in 2022 and sporadic outbreaks in New York and Gaza highlight the ongoing risk of polio, alongside the persistent global threat posed by wild-type poliovirus. This study aims to develop and validate a quantitative reverse transcription PCR (RT-qPCR) panel that employs different primer–probe sets to simultaneously detect vaccine and wild-type poliovirus (WPV) in wastewater. Using an inactivated poliovirus vaccine (IPV) and engineered DNA fragments (eDNAf), the qPCR master mix (MM) performance, assay specificity, sensitivity (limit of detection, LOD), and recovery from IPV-spiked wastewater were evaluated. Compared with two-step RT-qPCR and qScript MM, one-step RT-qPCR with TaqMan MM improved sensitivity for the following polioviruses (PV): Sabin 1 in IPV and the eDNAf of Sabin 1, 2, and 3; WPV1 and WPV3; and poliovirus type 2 (any serotype 2). The LOD for Sabin 1 in IPV was 2.49 copies/PCR, while LODs for eDNAf of polio targets ranged from 1.06 to 3.12 copies/PCR. Sabin 1 recovery from IPV-spiked wastewater ranged from 10.26% to 57.27%. The RT-qPCR panel for poliovirus exhibited good specificity and sensitivity, with moderate viral recovery, enabling rapid implementation of wastewater monitoring for PV as needed.

## 1. Introduction

The poliovirus surveillance network, which identifies clinical poliovirus infections and monitors the recurrence of viral signals in environmental samples (e.g., sewage), has remained a pivotal tool in efforts towards global polio eradication since the inception of the Global Polio Eradication Initiative (GPEI) in 1988 [1]. Three serotypes of wild-type PV (WPV1, WPV2, and WPV3), belonging to species C of *the Enterovirus* genus, family *Picornaviridae*, cause poliomyelitis, which affects the central nervous system and causes acute flaccid paralysis (AFP) predominantly in infected children, potentially resulting in lifetime disability and in some instances death [2]. In the pre-vaccine era, AFP was a common symptom of polio following infection, occurring mainly in children under 15 years old, although AFP may affect individuals of any age group [3]. Polio incidence declined significantly with the introduction of the Salk inactivated polio vaccine (IPV) [4] and the Sabin Oral Polio Vaccine (OPV) [5] in 1954 and 1957, respectively. Annual reported clinical cases of paralytic poliomyelitis decreased from 25,711 in 1988 to 3304 in 1995 in South-East Asia alone [6], representing an 87% reduction [7]. WPV2 was declared globally eradicated in September 2015 [8] following the last reported case in India in 1999. However, polio remains endemic in Afghanistan and Pakistan, with a few hundred cases of WPV1 each year [9]. The anti-polio vaccination programs of the GPEI are now facing challenges from clusters of paralytic polio cases emerging in under-immunized communities where live-attenuated OPV has been used [10]. Since April 2016, the trivalent oral poliovirus vaccine has been replaced by a bivalent oral poliovirus vaccine that contains only attenuated viruses of serotypes 1 and 3 for routine immunization [11]. In 2022, a number of AFP cases caused by circulating vaccine-derived poliovirus type 2 (cVDPV2) in Africa and Southeast Asia [12] and a single case in New York [13] underscore the risk of illness derived from VDPV. Substitution with IPV could prevent VDPV polio caused by live-attenuated OPV; however, costs, logistics, and demands are much higher [14].

In the 1940s, Melnick [15] and others [16] identified risks associated with poliovirus transmission in sewage, providing early evidence that wastewater monitoring might complement clinical diagnosis. Wastewater-based surveillance (WBS) has been used to detect sporadic AFP caused by VDPV, including the 2022 paralytic polio case in New York, where poliovirus was first detected in wastewater [13]. WBS has also demonstrated that poliovirus can circulate in sewage even in the absence of paralytic cases [17], including evidence of silent WPV1 transmission in Israel in 2013 [18]. The unprecedented COVID-19 pandemic has enriched scientific evidence on WBS as a valuable complementary tool to monitor viral pathogens of global concern at the community level [19,20,21].

Several rapid simplex [22,23] and multiplex [24,25] PCR-based detection methods have been reported and utilized for the identification of different serotypes of polioviruses. However, these simplex assays have been reported to detect either Sabin vaccine strains or WPV strains as individual RT-qPCR assays. Variations in thermocycling conditions, master mix, and degenerate primers impede the capacity for detecting both Sabin and WPV strains using a standardized simplex RT-PCR assay. To address this gap, we aimed to develop a rapid, reliable, and accurate RT-qPCR panel assay capable of simultaneously detecting and differentiating PV targets, including vaccine-derived Sabin (1, 2, and 3), WPV1 and 3, and PV2 (any serotype 2). The key innovations of this study are: (i) consolidation of six simplex assays into a single one-step RT-qPCR panel under unified thermocycling conditions; (ii) optimization of master mix and primer–probe sets to improve assay compatibility across Sabin and wild-type targets; and (iii) laboratory validation of specificity, sensitivity, and wastewater spike-recovery performance to support poliovirus wastewater monitoring. The specific purpose of the assay development is to enable immediate WBS monitoring of polioviruses that have the potential to cause an outbreak, thereby serving as a critical preparedness measure when public health authorities issue a call for an outbreak investigation.

## 2. Materials and Methods

### 2.1. Positive Controls

**Figure 1:**
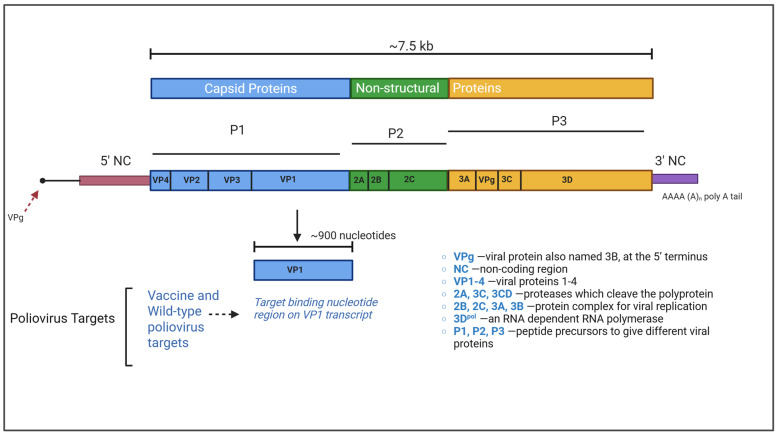
Poliovirus genome organization. *Created in BioRender. Immaraj, L. (2026) https://BioRender.com/oj14sd7* (accessed on 18 March 2026).

Inactivated polio vaccine (IPV) (Sanofi Pasteur, Toronto, ON, Canada) containing formaldehyde-inactivated Type 1 (Mahoney), Type 2 (MEF-1), and Type 3 (Saukett); and engineered DNA fragments (eDNAf) of Sabin (1, 2, and 3), WPV1, PV2 (any serotype 2), and WPV3 strains were used as positive controls to evaluate the RT-qPCR panel performance. The single-dose IPV vial (0.5 mL) contains 40, 8, and 32 d-antigen units (d-AU) of Type 1, 2, and 3 strains, respectively. Aliquots of 100 µL were stored in a −80 °C freezer. The engineered DNA fragments (eDNAf) synthesized by IDT (Integrated DNA Technologies, Coralville, IA, USA) contain the targeting sequences in the poliovirus viral protein 1 (VP1) region with 300–400 base pairs (bp) for the Sabin 1, 2, and 3, and WPV1, PV2, and WPV3, respectively (Figure 1). Each lyophilized eDNAf was resuspended in 20 µL of sterile RNase-free water at a final concentration of 10 ng/µL. A series of 10-fold dilutions of eDNAf, ranging from 10^−1^ to 10^−20^, was prepared in carrier RNA solution (10 µg/mL) and stored in single-use aliquots at −80 °C. To calculate absolute copy numbers, digital PCR assays (ThermoFisher Scientific, Burlington, ON, Canada) were used to quantify both vaccine and WPV targets in IPV and eDNAf.

### 2.2. Extraction of RNA

RNA was extracted from IPV (100 µL) using the MagMAX™-96 viral RNA isolation kit (ThermoFisher Scientific, Burlington, ON, Canada) with the automated KingFisher™ Flex system (ThermoFisher Scientific, Burlington, ON, Canada) according to the manufacturer’s protocol. The extracted RNA was eluted in a final volume of 50–100 μL of sterile RNase-free water, and a series of 10-fold dilutions ranging from 10^−1^ to 10^−6^ was prepared in carrier RNA solution (ThermoFisher Scientific, Burlington, ON, Canada) and stored in single-use aliquots at −80 °C.

### 2.3. Primers and Probes Used for the Detection of Poliovirus Strains

A combination of primer and probe sequences, as described in different groups previously [23,25,26,27], was tested for each vaccine-derived and WPV (1, 2, and 3) strains for the RT-qPCR panel assay. The specific primer sequences target the unique VP1 capsid region for each wild-type and vaccine strain. The eDNAf contained the VP1 target regions used for assay evaluation, and the corresponding primer–probe sequences, genomic locations, and source references are provided in Table 1.

Gerloff and Sun et al. primers and probes (2018) [26,27] included Sabin (1, 2, and 3), Pan PV (specific to any poliovirus), WPV1, PV2 (any serotype 2), and WPV3. Primers for the WPV1 and WPV3 strains target genotypes specific to wild poliovirus, African/West African (AFR-WEAF), and wild poliovirus, South Asia (SOAS) (Table 1). Additional assays from Manukyan and Sharma et al. (2018) [23,25] included Sabin 2, 3, WPV1-Sharma, and WPV3-Sharma. Custom primers and probes were also designed for eDNAf specific to the PV2 target (Table 1). All primers and probes used to develop the RT-qPCR panel assay were synthesized by IDT (Integrated DNA Technologies, Coralville, IA, USA or ThermoFisher Scientific, Burlington, ON, Canada), and preparations were stored at −20 °C.

### 2.4. Comparison of One-Step and Two-Step RT-qPCR Amplification

To assess whether the reverse transcription (RT) step influenced the sensitivity of the qPCR assay, RNA extracted from IPV and eDNAf of Sabin (1, 2, and 3), WPV1, PV2, and WPV3 were used in both one-step and two-step RT-qPCR assays. The one-step RT-qPCR reaction contained 2.5 μL of 4× Taqman Fast Virus one-step RT-PCR MasterMix (ThermoFisher Scientific, Burlington, ON, CA), 1 μL of primer–probe mixture (800 nM each of forward and reverse primer along with 200 nM probe), and 5 μL of template (IPV RNA) in a total volume of 10 μL. The thermocycling conditions were set to a standard run program and consisted of an RT step at 50 °C for 30 min and incubation at 95 °C for 1 min, followed by 40 cycles of PCR amplification at 95 °C for 15 s, 50 °C for 1 min, and elongation at 72 °C for 5 s. As previously described [26], a reduced ramp rate of 25% was applied between the annealing and elongation steps on the ABI instrument. For the two-step qPCR (RT and qPCR) assay [28], initially, 5 μL of RNA was pre-heated at 95 °C for 5 min and quick-chilled on ice for 5 min. The treated RNA was added to the 15 μL of RT-master mix containing 4 μL of 5 x first-strand buffer, 5 mM DTT, dNTPs (2.5 mM each of dATP, dCTP, dGTP, and dTTP), 600 ng of random primer, 20 units of RNaseOut™ recombinant ribonuclease inhibitor, and 100 units of SuperScript™ II reverse transcriptase enzyme. The total 20 μL mixture was incubated at 42 °C for 1 h, followed by 72 °C for 15 min. The transcribed cDNA was either aliquoted and stored at −20 °C for later use or proceeded with qPCR reaction containing a total volume of 10 μL: 5 μL of 2× TaqMan Fast Universal MasterMix (ThermoFisher Scientific, Burlington, ON, Canada), 2 μL of specific primer–probe mixture (900 nM each of forward and reverse primer along with 250 nM probe), and 3 μL of transcribed cDNA. Thermocycling conditions were set similarly to the one-step qPCR assay as described above, with the RT step removed. Both qPCR assays were performed on an Applied Biosystems (ABI) 7500 Fast PCR instrument (ThermoFisher Scientific, Burlington, ON, Canada). Comparative results determined the one-step RT-qPCR assay as the optimized assay based on cycle threshold (Ct) evaluation; therefore, this assay was used to assess the remaining validation parameters described below. Final optimized digital PCR quantification of each control used for validation is reported in Table 2.

### 2.5. Evaluation of Different qPCR Master Mixes on RT-qPCR Amplification

In previous studies [26,27], the qScript XLT one-step RT-qPCR ToughMix was used for RT-qPCR detection of PV targets. Hence, we compared the qScript MM with our routinely used TaqMan MM to evaluate the detection sensitivity of the PV targets in the RT-qPCR assays. A series of 10-fold dilutions of the positive controls, IPV and eDNAf (Sabin 1, 2, and 3, Pan PV, WPV1, PV2, and WPV3), were tested using two PCR master mixes **A:** A 4 × TaqMan Fast Virus one-step RT-PCR MasterMix (ThermoFisher Scientific, Burlington, ON, CA) and **B:** qScript XLT one-step RT-quantitative PCR (qPCR) ToughMix [26] (Quanta Biosciences, Beverly, MA, USA). The RT-qPCR assays were performed using the one-step PCR thermocycling conditions on the ABI 7500 Fast PCR instrument, and cycle threshold (Ct) values, amplification curves, and multicomponent plots were evaluated. Extraction-negative and non-template negative controls (carrier RNA or nuclease-free water) were included in all RT-qPCR assays as quality controls. Samples exhibiting smooth sigmoidal amplification, raw fluorescence curves, and good Ct values were considered ‘true positive.’ Conversely, samples lacking a product signal, exhibiting flat amplification plots, and yielding undetermined Ct values were deemed negative. Amplification curves were analyzed with thresholds set at 0.05 for TaqMan assays and 10,000 for qScript assays.

### 2.6. Determination of Specificity, Sensitivity, and Precision of One-Step RT-qPCR for Detection of Poliovirus Targets

The specificity of the one-step RT-qPCR assay was determined by optimizing various primers and probes to test the PV targets in IPV or eDNAf for optimal amplification and detection under identical thermocycling conditions. Cross-reactivity testing was conducted using clinical stocks of different viral strains at medium to high viral loads; this was done to determine whether the PV targets in IPV and eDNAf were specific and if they cross-reacted with other enteroviruses, such as coxsackievirus B3 and echovirus 9, as well as with respiratory viruses such as severe acute respiratory syndrome coronavirus 2 (SARS-CoV-2) N1/N2 genes, different human coronavirus (HCoV) strains (229E, OC43), influenza A (H1N1/H3N2) and B, and respiratory syncytial virus (RSV-L) given the assay’s intended use for WBS. The sensitivity of our assay was evaluated by the limit of detection (LOD) of each PV target using 10-fold serial dilutions of the positive controls: IPV (Sabin 1—1.92 × 10^5^ copies) and eDNAf (Sabin 1—1.83 × 10^5^, Sabin 2— 2.06 × 10^5^, Sabin 3— 7.29 × 10^4^, WPV1— 7.21 × 10^4^, PV2—1.35 × 10^4^, and WPV3— 5.11 × 10^4^ copies). At each dilution, 10 replicates were analyzed, and the 95% LOD was calculated by probit logistic regression analysis, as described previously [29]. Standard curves were plotted using Ct values against log copies per PCR reaction, and the amplification efficiency was assessed by standard curve parameters, such as the slope and the coefficient of determination (R^2^). The precision of RT-qPCR was analyzed using the inter-variability of Ct values obtained from replicates of the standard curve performed on two different days.

### 2.7. Evaluation of Polio Targets Recovery

The recovery rate of Sabin 1 from IPV-spiked wastewater was evaluated by adding 100 µL of IPV (1.92 × 10^5^ copies) to 100 mL of raw wastewater samples (*n* = 3). Baseline control samples were prepared by adding 100 µL of the same IPV aliquot to 900 µL of PBS. Wastewater samples were processed and concentrated as previously described [30,31], and both the liquid and solid fractions were retained. RNA extracts were prepared from both fractions as described in Section 2.2. Human coronavirus 229E (HCoV-229E) was purchased from the ATCC (VR-740) and propagated in MRC-5 cells, and aliquots of the virus stock were stored at −80 °C. During RNA extraction, HCoV-229E (4.8 × 10^5^ IU/mL) was spiked into the lysis buffer for the baseline and wastewater samples as an internal process control to monitor extraction performance and matrix-associated effects. Spiked and baseline samples were tested in parallel using the validated one-step RT-qPCR conditions. The percentage of recovery (%) was calculated as:

Percentage of recovery (%) = the amount of target detected in spiked test sample/the amount of target detected in baseline sample × 100

### 2.8. Statistical Analysis

All data are represented as Mean ± SD unless otherwise specified. Standard curve plotting and statistical analysis were performed using GraphPad Prism (version 10.6.1). The 95% LOD was calculated by probit logistic regression analysis, as described previously [29]. 

## 3. Results

### 3.1. One-Step vs. Two-Step RT-qPCR Assays

One-step and two-step RT-qPCR assays were used to detect PV targets, including Sabin 1, Sabin 2, Sabin 3, Pan PV, WPV1, PV2, and WPV3 (AFR and SOAS) in IPV. Compared to the one-step assay, the two-step assay increased the Ct value by 4 for Sabin 1 (from 14.55 ± 0.81 to 18.18 ± 0.23) and 6 for Pan PV (from 25.39 ± 3.32 to 31.35 ± 0.38), indicating that the two-step approach did not improve the sensitivity of the qPCR detection. In addition, no detection was observed for Sabin 2 (FAM), Sabin 3 (VIC), WPV1, PV2, and WPV3 (AFR and SOAS) targets in IPV using either method. The two-step RT-PCR assay did not perform better than the one-step assay.

### 3.2. Evaluation of PCR Master Mixes on Detection Sensitivity and Precision of Poliovirus Strains

(a) IPV

**Figure 2:**
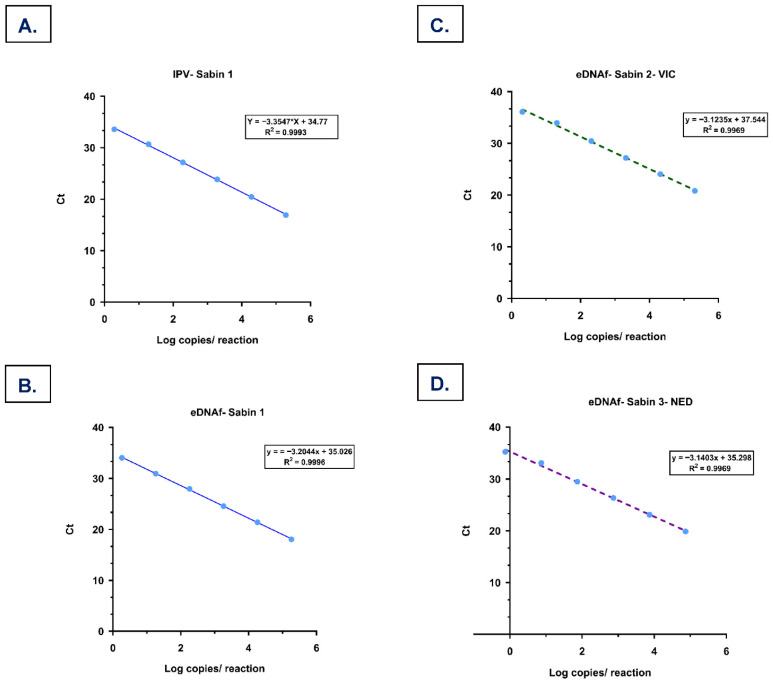

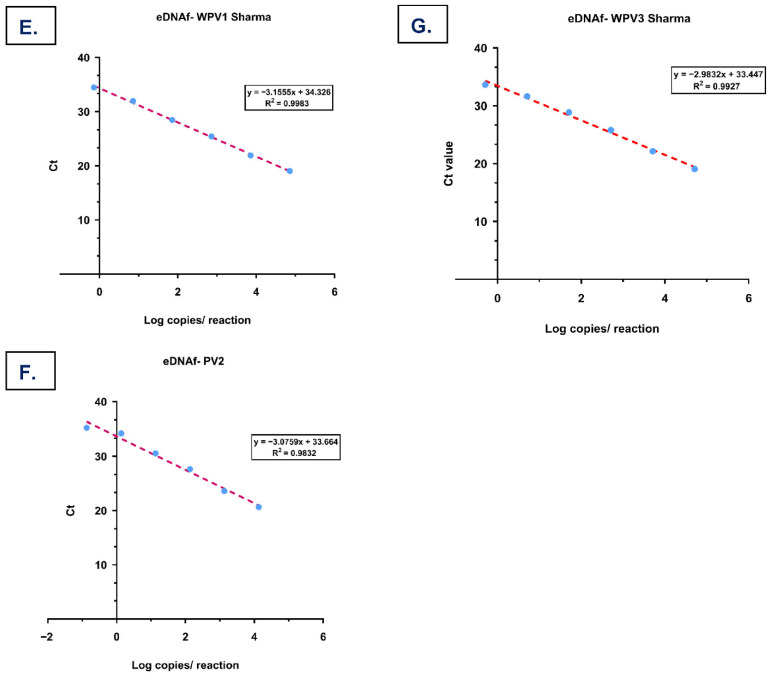
Standard curves of (**A**) IPV and eDNAf (**B**–**G**) used for quantification in one-step RT-qPCR for PV targets: (**B**) Sabin 1, (**C**) Sabin 2, (**D**) Sabin 3, (**E**) WPV1, (**F**) PV2, and (**G**) WPV3.

One-step RT-qPCR using the TaqMan MM showed good sensitivity for Sabin 1 in IPV, with quantification ranging from 1.92 × 10^5^ to 1.92 × 10^0^ copies per PCR reaction. A mean Ct difference of 3.33 ± 0.11 was observed between the 10-fold serial dilutions (Table 2), and the standard curve parameters showed a slope and R^2^ at −3.35 and 0.99, respectively (Figure 2A). Reduced detection sensitivity and higher standard deviations were observed using the qScript MM (Ct range 21.07 ± 0.21 to 34.76 ± 0.77) compared to the TaqMan MM.

Sabin 2 primers and probes were not detected in IPV using the TaqMan and qScript master mixes. Similarly, Sabin 3 with the VIC probe was not detected in IPV using the TaqMan MM; however, late amplification curves with high Ct values (39.49 ± 0.20) were observed using the qScript master mix. In contrast, Sabin 3 with the NED probe showed amplification detections with high Ct values (35–39) using both the TaqMan and qScript master mixes. Due to inconsistent detection of Sabin 2 and Sabin 3 in IPV, characterized by absent or delayed amplification and high Ct variability across primer–probe sets and master mixes, only Sabin 1 was included in further optimization stages of the RT-qPCR panel.

The Pan PV assays showed moderate detection sensitivity and precision in IPV with the qScript MM compared to the TaqMan MM. The Ct values ranged from mid to high, between 26.02 ± 0.54 and 39.38 ± 0.02 for the Pan PV-FAM assay and 27.42 ± 1.34 to 41.41 ± 0.01 for the Pan PV-ZEN assay. Although a good detection range and moderate sensitivity were observed with the Pan PV assays using the qScript MM, these assays were excluded from the RT-qPCR panel for three reasons: (1) the TaqMan MM did not improve detection sensitivity for Pan PV target using FAM or ZEN probes; (2) ‘crooked or distorted’ amplification plots were obtained using the qScript MM; and (3) the raw fluorescence curves stayed close to flat lines across all reactions on the multicomponent plots and did not display healthy tall sigmoidal curves, typical of a positive detection. In addition, upon testing the wild-type strain-specific targets WPV1, PV2, AFR-WPV3, and SOAS-WPVS in IPV using the TaqMan MM, no detection was observed, suggesting the IPV only contains the vaccine strains, which are specific and do not cross-react with the wild-type strains.

(b) eDNAf

Greater sensitivity and precision were observed with the TaqMan MM at different concentrations compared to the qScript MM for each of eDNAf: Sabin 1 (Cy5), Sabin 2 (VIC), and Sabin 3 (NED) (Table 2), suggesting that the TaqMan MM improved the sensitivity and precision of the qPCR assay for PV target detection.

We also tested the eDNAf using primers and probes specific to wild-type strains (WPV1, PV2, AFR WPV3, and SOAS WPV3) [26] with the TaqMan and qScript MM. WPV1 and PV2 were not detected using either of the master mixes. AFR WPV3 showed good detection sensitivity using qScript MM, while SOAS WPV3 exhibited diminished detection performance with both the master mixes. Since the WT targets, WPV1-Sharma (7.21 × 10^4^ to 7.21 × 10^−1^ copies), PV2 (1.35 × 10^4^ to 1.35 × 10^−1^ copies), and WPV3-Sharma (5.11 × 10^4^ to 5.11 × 10^−1^ copies), demonstrated better sensitivity, precision, and amplification curve quality, these targets were further validated in contrast to the previously tested wild-type 1, 2, and 3 strains. After evaluating both the TaqMan and qScript master mixes and testing different combinations of primers and probes, the RT-qPCR panel was finalized to include PV targets specific to Sabin 1 [26] in IPV, and Sabin 1 [26], Sabin 2 and 3 [25], WPV1-Sharma [23], PV2, and WPV3-Sharma [23] in eDNAf (Table 3).

### 3.3. The Specificity and Sensitivity of One-Step RT-qPCR for the Detection of Poliovirus Targets

The RT-qPCR panel detected Sabin 1 in IPV, and the eDNAf of Sabin 1, 2, and 3, WPV1-Sharma, PV2, and WPV3-Sharma with no cross-reactivity observed. Across all specificity experiments, enteroviruses and respiratory viruses did not yield specific amplification signals in the poliovirus RT-qPCR assays, indicating that each assay was specific and that the panel could differentiate various Sabin and WPV strains without any off-target amplification. In IPV, as previously stated, good sensitivity was observed for Sabin 1, with Ct values ranging from 16.90 ± 0.13 to 33.58 ± 0.71, corresponding to 1.92 × 10^5^ to 1.92 × 10^0^ copy numbers. (Table 2). The 95% LOD of Sabin 1 in IPV was 2.49 copies/PCR reaction by probit analysis (Table 4). The efficiency of the qPCR assay was observed to be 99% from the standard curve (Figure 2A). The coefficient of variation (CV) for the Ct values generated from 10 replicates of RT-qPCR was determined to be 0.83% for 1.92 × 10^5^ copies/reaction and 2.11% for 1.92 × 10^0^ copies /reaction, respectively.

The 95% LOD for the eDNAf of Sabin 1, 2, and 3 was 2.07, 3.12, and 1.12 copies/PCR reaction, respectively (Table 4). For the eDNAf of wild-type targets, WPV1-Sharma, PV2, and WPV3-Sharma, the 95% LODs were 1.06, 2.03, and 1.11 copies/PCR reaction, respectively, with qPCR efficiencies of both Sabin and WPV targets ranging between 105 and 116% (Figure 2B–G). The CV for the Ct values obtained at lower concentrations of eDNAf, Sabin 1, 2, and 3, WPV1, PV2 and WPV3 were 0.92%, 3.0%, 4.1%, 2.7%, 1.8%, and 2.5%, respectively. Negative controls for all RT-qPCR assays presented no amplification products with undetermined Ct values.

### 3.4. Recovery of Poliovirus Targets from Wastewater

Sabin 1 recovery was mainly observed in the liquid fractions of spiked wastewater samples. Across three biological replicates, Sabin 1 recovery ranged from 10.26% to 57.27% (Table 5), with corresponding Ct values of 18.08 ± 0.21 to 20.58 ± 0.13 relative to baseline controls (Ct, 17.26 ± 0.04). In contrast, Sabin 1 recovery in the solid fractions was trace (≤0.01% in neat solids) and remained low after serial dilutions (≤0.45%), suggesting limited solids-associated distribution under the tested conditions. In the liquid fractions, hCoV-229E Ct values were comparable with the baseline (26.58 ± 0.67 vs. 26.22 ± 0.03; mean Ct difference = 0.36). In the solid fractions, hCoV-229E Ct values were increased (29.49 ± 0.16; mean Ct difference = 3.27), consistent with reduced amplification performance in the solid fraction.

## 4. Discussion

Poliovirus transmission in London [32], Israel [33], and the USA [13] serves as a stark reminder that no country is immune to the threat of polio until global eradication is achieved. Recently, sporadic outbreaks of poliovirus infection in children have been reported increasingly, specifically in war- and conflict-prone areas [34], underscoring the ongoing risk posed by revertant and highly transmissible poliovirus strains. Given its significant and long-lasting morbidity, clinical surveillance remains essential for monitoring PV across borders [35], with WBS serving as a complementary approach. The global campaign for anti-polio vaccination still remains highly demanding [36]. WBS for polioviruses has played a crucial role in the past and is now poised to play an important role with the resurgence of clinical cases [33]. WBS can enable early detection of polioviruses before symptomatic cases are confirmed, help assess the types of circulating strains, differentiate VDPV from WPV to inform possible transmission risk, and monitor the genetic evolution of polioviruses at a community level.

Numerous molecular assays to detect and quantify poliovirus have been reported, yet reproducibility remains inconsistent. In this study, we assessed six different complementary sets of primers and probes and validated their use under universal thermocycling conditions, enabling the simultaneous detection of various PV targets, including vaccine-derived (Sabin 1, 2, and 3) and wild-type (WPV 1, PV2, and WPV3) strains, for application in wastewater samples. Compared to the two-step RT-qPCR approach, which showed a delay of four to six cycles in detecting Sabin 1 and Pan PV targets in IPV, the one-step RT-qPCR assay showed greater sensitivity. This observation was consistent with previous reports, which used one-step RT-qPCR [22,23,24] or one-step multiplex RT-qPCR [25] methods to detect PV targets in wastewater samples. However, some limitations were reported, such as the inability to detect WPV and Sabin strains simultaneously in a single RT-qPCR panel.

To further improve the newly developed one-step RT-qPCR panel assay, we optimized the master mix for the assay. The TaqMan MM enhanced sensitivity and precision for detecting Sabin 1 in IPV. However, both master mixes were unable to detect Sabin 2 but detected Sabin 3 (NED) at high Ct values, suggesting that current IPVs contain low quantities of Sabin 2 and Sabin 3 strains, which predominantly constitute the OPVs [37]. IPV is conventionally prepared from formalin-treated virulent poliovirus strains (Mahoney, MEF-1, and Saukett for types 1, 2, and 3, respectively). We used the Sabin strains in IPV for two main reasons: (i) non-availability of OPV for testing and (ii) the fact that, historically, the Sabin 1 strain was derived from the Type 1 (Mahoney) strain present in IPV [38,39], indicating similarities between the two strains. IPVs and OPVs exhibit distinct antigenic and immunogenic properties [37]. Our results showed that the one-step RT-qPCR assay was more effective at detecting Sabin 1 in IPV than Sabin 2 or Sabin 3. Wild-type strains WPV1, PV2, AFR-WPV3, and SOAS-WPV3 were not detected in IPV using primers and probes from Gerloff et al. [26], indicating no cross-reactivity between the vaccine and wild-type strains. The Pan PV (any poliovirus) assays using FAM and ZEN probes exhibited better sensitivity with the qScript MM than with the TaqMan MM in IPV. The updated Pan PV assay with ZEN double quencher [27] showed improved detection sensitivity compared to the standard Pan PV-FAM assay at lower IPV concentrations. Due to moderately high Ct values (26 to 30) at higher IPV concentrations, inter-variability between qPCR runs, distorted amplification plots, and flat fluorescence signals led us to exclude both Pan PV assays from the RT-qPCR panel.

The improved detection sensitivity of the one-step RT-qPCR assay was also observed with the TaqMan MM compared to the qScript MM when eDNAf specific to Sabin 1, 2, and 3 vaccine strains were used for evaluation, although Sabin 2 and Sabin 3 showed a better limit of detection (LOD) with the Manukyan et al. [25] primers and probes. No detection was observed for both WPV1 and PV2 targets in eDNAf using the Gerloff et al. [26] primers and probes. Detection of WPV3 targets, AFR-WPV3, and SOAS-WPV3 varied by master mix: the qScript MM showed improved sensitivity for AFR-WPV3, while SOAS-WPV3 performed poorly with both master mixes. However, inter-variability between replicates was observed. Notably, the Gerloff et al. [26] primer/probe sets effectively detected Sabin strains using the TaqMan MM and did not enhance detection for the WT strains. Our results indicated that pairing the TaqMan MM with the Sharma et al. [23] primers for WPV1 and WPV3, along with a custom PV2 primer–probe set, significantly enhanced wild-type poliovirus detection in the one-step RT-qPCR assay. Previous studies have demonstrated that the TaqMan MM paired with non-degenerate primers improved sensitivity in the qPCR detection of polio gene targets in polio-inoculated cell cultures [22,23]. The use of modified degenerate primers with purine or pyrimidine nucleosides, mixed-base, and inosine residues improves the specificity of qPCR assays, although this approach may compromise the sensitivity of detection [40]. The degenerate PV primers were designed and validated for qPCR amplification of plaque-purified PV targets grown in cell cultures [26]. The use of the Gerloff et al. [26] WT primer–probe sets with the degenerate bases could explain the poor sensitivity in detecting WPV1, PV2, AFR WPV3, and SOAS WPV3 in eDNAf in this study. The one-step RT-qPCR assay panel demonstrated enhanced sensitivity and precision for detecting both Sabin and wild-type poliovirus targets in eDNAf when the TaqMan MM was paired with optimized primers from Gerloff, Manukyan, and Sharma et al., emphasizing the importance of master mix–primer–probe compatibility for optimal assay performance.

The 95% LOD for Sabin 1 in IPV was 2.49 copies/ reaction. In eDNAf, the 95% LOD for Sabin and WPV strains ranged between one and three copies per reaction, consistent with previous findings [24,25,26]. Excellent reproducibility was also observed for all six PV targets, including Sabin 1, 2, and 3, as well as WPV 1-Sharma, PV2, and WPV3-Sharma. The efficiency of the PV assays for the different targets ranged from 105 to 116%. All PV targets evaluated in this study were specific and did not cross-react with other enterovirus and respiratory viral pathogens. Viral recovery from wastewater samples is another critical step for the detection of poliovirus. Numerous studies [41,42] have reported consistent recovery after spiking viral targets into wastewater matrices. Recovery rates can vary with wastewater matrices, processing volume, viral size, and the concentration method used, all of which can affect downstream detection [43,44,45]. Typically, higher concentrations of viral targets in wastewater are associated with improved detection and recovery rates [44,45]. In our study, Sabin 1 recovery from IPV-spiked wastewater was primarily observed in the liquid fraction, with recovery ranging from 10.26% to 57.27% (Table 5). These results demonstrate measurable recovery of poliovirus from the aqueous phase using our routine workflow, supporting the feasibility of detecting low levels of poliovirus in wastewater. Recovery in the solid fraction was trace and remained low after serial dilutions, indicating limited solid-associated Sabin 1 signal under the conditions tested in this study. As an internal control added during extraction, hCoV-229E showed a minimal Ct shift in the liquid fraction relative to baseline controls (mean Ct difference = 0.36), whereas a larger Ct increase (3.27) was observed in the solid fraction, consistent with reduced overall assay performance in solids. The minimal Ct difference in the liquid fraction suggests comparable extraction and RT-qPCR performance to the baseline, whereas the larger Ct increase in the solid fraction indicates reduced recovery or amplifiable template in solid-derived extracts. Thus, these findings indicate that the solid matrix performed poorly overall compared to the liquid matrix under the tested conditions, consistent with trace detection of Sabin 1 in solids.

Compared to previously published RT-qPCR assays, our panel offers advantages for laboratories implementing poliovirus WBS. Multiplex RT-PCR methods, such as the assay by Manukyan et al. [25], were designed for high-throughput quantification of the three Sabin vaccine strains in clinical or vaccine-derived specimens and do not include wild-type polioviruses within a single panel [25]. Similarly, other RT-qPCR assays target either vaccine or wild-type strains and require different master mixes and thermocycling conditions for the detection of individual PV targets [22,23,24,25,26]. In contrast, we consolidated six simplex assays into a single one-step RT-qPCR panel, demonstrating high sensitivity and specificity, reproducibility, and moderate recovery suitable for deployment in wastewater samples.

**Figure 3:**
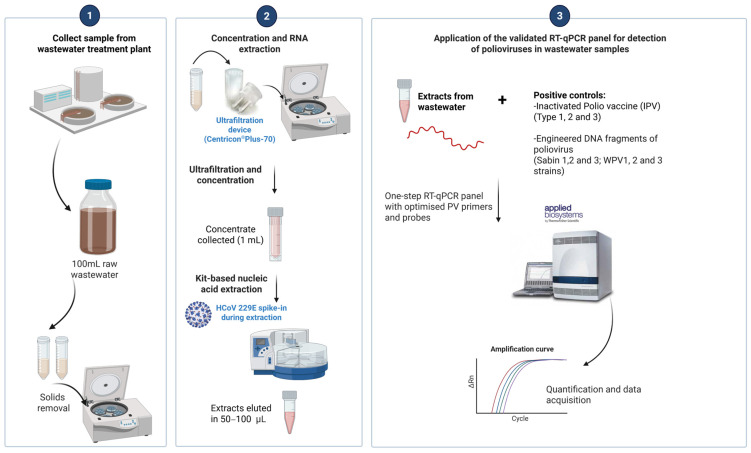
Illustration of a rapid, specific, and sensitive RT-qPCR panel for simultaneous detection and differentiation of poliovirus targets for application to wastewater samples, based on laboratory-validated wastewater concentration and extraction protocol [31,46]. *Created in BioRender. Immaraj, L. (2026) https://BioRender.com/7c0wwqz* (accessed on 18 March 2026).

This study had several notable limitations. Firstly, testing for vaccine-derived PV was confined to IPV, which does not contain high titers of Sabin 2 and Sabin 3. The oral polio vaccine (OPV) containing Sabin strains was not available. Secondly, there was no possible access to PV-positive clinical specimens for this evaluation; therefore, we were unable to compare the specificity and sensitivity of the RT-qPCR panel when applied to PV clinical samples. Thirdly, the recovery and amplification performance varied depending on the fraction. Sabin 1 recovery was primarily observed in the liquid phase, with only trace amounts in solids. Since our routine workflow (Figure 3) focuses on the liquid fraction after the removal of solids, solid-associated recovery was assessed only to provide a complete recovery assessment across both fractions. Given the relatively low viral titers of Sabin strains in IPV, future evaluations using OPV containing higher titers of live-attenuated virus may yield improved recovery rates, thereby enhancing the practical applicability of the assay in routine WBS.

In conclusion, we have optimized and validated a simplex one-step RT-qPCR panel assay using universal thermal cycle conditions and a matched master mix to simultaneously detect and identify the full range of polioviruses in wastewater samples. This panel is highly sensitive for detecting Sabin and WPV strains in IPV and eDNAf. The panel assay represents a promising tool for poliovirus wastewater monitoring and warrants further evaluation using community wastewater samples in real-world outbreak settings. Amidst continuing threats of sporadic poliovirus infection and polio-associated diseases, this RT-qPCR panel undoubtedly provides another valuable and complementary tool for monitoring the diverse poliovirus strains circulating in a community, as an essential step for potential outbreak preparedness.

## Figures and Tables

**Table 1:** Primers and probe sequences used for optimization and identification of poliovirus target genes in the RT-qPCR panel assay.

Polio Strain Type	Virus Strain	Primer and Probe Names	Sequence 5′→3′ (Reference)	Genomic Location	Amplicon Size (bp)	Ref. No.
Sabin 1 (vaccine)	Sabin 1	Sabin 1 2S Sabin 1 1A Sabin 1 probe A4	AGG TCA GAT GCT TGA AAG CCGC CCA CTG GCT TCA GTG TTT Cy5-CCC CAC CGT TTC ACG GA-BHQ3	2505–2523 2600–2583 2540–2559	95	[26]
Sabin 2 (vaccine)	Sabin 2	Sabin 2 2S Sabin 2 1A Sabin 2 probe	CCG TTG AAG GGA TTA CTA AA CGG CTT TGT GTC AGG CA FAM-ATT GGT TCC CCC GAC TTC CAC CAA T-BHQ1	2525–2544 2595–2579 2550–2572	70	[26]
	Sabin 2	2682TqS2F 2803TqS2R Sab2	CCAGAGACGAACGCGA CAAACCGAAAACAATCTGC VIC-CACGGTTGAGTCATTC-NFQ	2688–2703 2810–2792 2712–2727	122	[25]
Sabin 3 (vaccine)	Sabin 3	Sabin 3 2S Sabin 3 1A Sabin 3 probe	AGG GCG CCC TAA CTT T TTA GTA TCA GGT AAG CTA TC VIC-TCACTCCCGAAGCAACAG-TAMRA	2537–2552 2591–2572 2554–2571	54	[26]
	Sabin 3	1411TqS3F 1629TqS3R Sab3	GGGAAAATTTTACTCCCAA TGAATCAATGGCCAAAGCA NED-AACGCAGTAACATCC-NFQ	1419–1437 1617–1599 1450–1464	199	[25]
Pan PV (any poliovirus)	Pan PV (any poliovirus)	Pan PV/PCR-S1 Pan PV PCR-1A Pan PV PCR-probe 1A	TTG GAG TTC TTC ACI TAI TCI MGI TTY GAY ATG GGA GCT CCG GGT GGG AYR TAC ATI ATY TGR TAI AC FAM-TGR TTN ARI GCR TGI CCR TTR TT-BHQ1	2832–2864 2962–2928 2926–2904	130	[26]
	Pan PV (updated)	Pan PV/PCR-S1 Pan PV PCR-1A Pan PV PCR-probe	TTG GAG TTC TTC ACI TAI TCI MGI TTY GAY ATG GGA GCT CCG GGT GGG AYR TAC ATI ATY TGR TAI AC FAM-TGR TTN ARI GCR TGI CCR TTR TT-Zen8	2832–2864 2962–2928 2926–2904	130	[27]
Wild-type Polio 1	WPV1 (Wild PV1)	WEAF WPV1 S SOAS WPV1 S WPV1 A WPV1 probe S	GTA CAA ACC AGT CAY GTN AT CGT ACA GAC TAG RCA YGT NAT GAG AAT AAY TTG TCY TTK GAY GT FAM-CAT WAT GGT TAC RCA MGC ACC T-BHQ1	2661–2680 2660–2680 2800–2778 2729–2750	139	[26]
	WPV1-Sharma	WPV1-F-Sharma WPV1-R-Sharma WPV1-PR-Sharma	AACAATGGGCATGCTTTGAAT TTTTCTGGCACTGGTGCG FAM-CAGGTCTATCAAATCAT-NFQ	436–456 506–489 457–473	71	[23]
Polio 2 (any serotype-wild-type or vaccine)	PV2 (any serotype 2)	PV Type 2 S PV Type 2 A PV Type 2 A 1C PV Type 2 probe S- PV Type 2 probe 1D S	GAT GCA AAY AAC GGI CAT GC TCA TAA AAG TGG GAR TAC GCR TT TCG TAA AAA TGA GAA TAT GCA TT FAM-ATG ACT ATA CGT GGC AGA C-BHQ1 FAM-CRC CKA TIC CTG GYA-BHQ1	2911–2930 3110–3088 3110–3088 2993–3011 2972–2986	199	[26]
	PV2 (any serotype 2)	PV2-F PV2-R PV2-probe	TCCAATTATACCGATGCAAACAA CCCCAGGTGGTATGTACATTATCTG VIC-CACGCACTAAATCAA-NFQ	2899–2921 2971–2947 2926–2940	73	Current study
Wild-type Polio 3	AFR WPV3 WPV3-I WEAF genotype	SOAS WPV3 S WEAF WPV3 S WPV3 A WPV3 probe S	CAG GGA GTA GAT GAY CTN AT CAG GGG GTT GAT GAY TTR AT ACK GTG TCT GAY GGN AC Cy5-CNC ARA ACA GYC TTC CGG ATA CC-BHQ3	2443–2462 2443–2462 2623–2607 2504–2526	180	[26]
	SOAS WPV3 WPV3-II SOAS genotype	SOAS 6S SOAS 5A SOAS WPV3 probe S	GTY RTA CAR CGR CGY AGY AGR A TCY TTR TAI GTR ATG CGC CAA G FAM-TTC TTY GCA AGI GGR GCR TGY GT-BHQ1	2671–2692 2816–2795 2713–2735	145	[26]
	WPV3-Sharma	WPV3-F-Sharma WPV3-R-Sharma WPV3-PR-Sharma	GGTGTTCTTGCTGTAAGAGTTGTGA CGCACCTTGGATGTAACTTTTG FAM-CGATCACAACCCC-NFQ	709–733 770–749 735–747	62	[23]

Specific probe labels are highlighted in green, and quenchers are highlighted in red. Cy5, Sulfo-Cyanine5; FAM, 6-carboxy-fluorescein; VIC, 2′-chloro-7′phenyl-1,4-dichloro-6-carboxy fluorescein; NED, 2-chloro-5-fluoro-7,8-benzo-1,4-dichloro-6-carboxy-fluorescein; BHQ, black hole quencher; TAMRA, Tetramethylrhodamine; Zen™, Iowa Black Quencher; NFQ, non-fluorescent quencher.

**Table 2:** Comparison of vaccine (Sabin) and wild-type (WPV) detections in IPV and eDNAf using two qPCR formats with various master mixes.

PV Targets	Quantified by One-Step RT-dPCR	1-Step RT-qPCR	
		4 × TaqMan MM	qScript XLT MM
	Copy/Reaction	Ct Value (Mean ± SD)	Ct Value (Mean ± SD)
IPV (Sabin 1-Cy5)	1.92 × 10^5^	16.91 ± 0.14	21.07 ± 0.21
	1.92 × 10^4^	20.45 ± 0.10	24.98 ± 0.15
Sabin 1-Cy5	1.83 × 10^6^	14.06 ± 0.52	19.90 ± 0.88
	1.83 × 10^5^	18.06 ± 0.41	23.37 ± 0.96
Sabin 2-VIC	2.06 × 10^6^	16.05 ± 0.01	24.92 ± 1.78
	2.06 × 10^5^	20.08 ± 0.30	26.87 ± 0.07
Sabin 3-NED	7.39 × 10^5^	16.24 ± 0.08	21.03 ± 0.71
	7.39 × 10^4^	19.88 ± 0.46	23.63 ± 0.39
WPV1-Sharma	7.21 × 10^5^	16.55 ± 0.52	UD
	7.21 × 10^4^	19.04 ± 0.58	UD
PV2	1.35 × 10^5^	16.55 ± 0.20	20.57 ± 0.39
	1.35 × 10^4^	20.80 ± 0.94	23.44 ± 0.07
WPV3-Sharma	5.11 × 10^5^	14.27 ± 0.64	UD
	5.11 × 10^4^	19.08 ± 0.90	UD

UD, Undetermined.

**Table 3:** Optimized primer–probe and master mix pairings for future polio assay implementation.

PV Targets	TaqMan MM	qScript MM	Preferred	qPCR Performance Summary	Primer/Probe Set (Ref. No.)
Sabin 1-Cy5	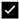	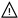	TaqMan	High sensitivity, reproducible (TaqMan) Inter-variability (qScript)	[26]
Sabin 2-VIC	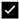	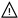	TaqMan	High sensitivity, reproducible (TaqMan) Inter-variability and low sensitivity (qScript)	[25]
Sabin 3-NED	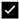	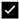	TaqMan	Sensitivity is comparable between the two master mixes, reproducible	[25]
WPV1-Sharma	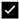	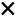	TaqMan	High sensitivity, reproducible (TaqMan) Discordant results (qScript)	[23]
PV2	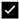	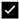	TaqMan or qScript	Sensitivity is comparable between the two master mixes, reproducible	Current Study
WPV3-Sharma	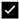	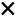	TaqMan	High sensitivity, reproducible (TaqMan) Discordant results (qScript)	[23]

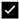
 = robust, 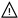 = use with caution, 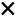 = not suitable for PV panel.

**Table 4:** Standard curve metrics and limit of detection (LOD) of PV targets.

PV Targets	Dynamic Range (Copies/Reaction)	Slope	Intercept	R^2^	Calculated Efficiency (%)	LOD_95%_ (Copies/Reaction)
IPV-Sabin 1 Cy5	1.92 × 10^0^–1.92 × 10^5^	−3.35	34.77	0.99	99	2.49
Sabin 1 Cy5	1.83 × 10^0^–1.83 × 10^5^	−3.20	35.03	0.99	105.15	2.07
Sabin 2 VIC	2.06 × 10^0^–2.06 × 10^5^	−3.12	37.54	0.99	109.01	3.12
Sabin 3 NED	7.39 × 10^−1^–7.29 × 10^4^	−3.14	35.30	0.99	108.18	1.12
WPV1 Sharma	7.21 × 10^−1^–7.21 × 10^4^	−3.16	34.33	0.99	107.45	1.06
PV2	1.35 × 10^−1^–1.35 × 10^4^	−3.08	33.66	0.98	111.40	2.03
WPV3 Sharma	5.11 × 10^−1^–5.11 × 10^4^	−2.98	33.45	0.99	116.38	1.11

**Table 5:** Sabin-1 recovery in IPV-spiked wastewater samples.

Sample No	Sabin 1			hCoV-229E
	Recovery (%)	Ct (Mean ± SD)	Baseline Ct (Mean ± SD)	Ct (Mean ± SD)
Sample 1	57.27	18.08 ± 0.21	17.26 ± 0.04	25.92 ± 0.06
				
Sample 2	37.46	18.69 ± 0.02	17.26 ± 0.04	26.54 ± 0.09
				
Sample 3	10.26	20.58 ± 0.13	17.26 ± 0.04	27.27 ± 0.03

## Data Availability

The original contributions presented in this study are included in the article. Further inquiries can be directed to the corresponding author.

## Abbreviations

The following abbreviations are used in this manuscript:

PV	poliovirus
AFP	acute flaccid paralysis
VDPV	vaccine-derived poliovirus
RT-qPCR	quantitative reverse transcription PCR
WPV	wild-type poliovirus
IPV	inactivated polio vaccine
eDNAf	engineered DNA fragments
MM	master mix
LOD	limit of detection
GPEI	Global Polio Eradication Initiative
OPV	oral polio vaccine
cVDPV2	circulating VDPV type 2
WBS	wastewater-based surveillance
d-AU	d-antigen units
IDT	Integrated DNA Technologies
VP1	viral protein 1
AFR	wild poliovirus African
WEAF	wild poliovirus West-African
SOAS	wild poliovirus South Asia
RT	reverse transcription
DTT	dithiothreitol
dNTPs	deoxynucleoside triphosphates
dATP	deoxyadenosine triphosphate
dCTP	deoxycytidine triphosphate
dGTP	deoxyguanosine triphosphate
dTTP	deoxythymidine triphosphate
Ct	cycle threshold
SARS-CoV-2	Severe acute respiratory syndrome coronavirus 2
HCoV	Human Coronavirus
RSV	Respiratory syncytial virus
Cy5	Sulfo-Cyanine 5
FAM	6-carboxy-fluorescein
VIC	2′-chloro-7′phenyl-1,4-dichloro-6-carboxy fluorescein
NED	2′-chloro-5′-fluoro-7′,8′-benzo-1,4-dichloro-6-carboxy-fluorescein
BHQ	black hole quencher
NFQ	non-fluorescent quencher
TAMRA	Tetramethylrhodamine
Zen-IBFQ	internal quencher- Iowa Black^®^ Fluorescent Quencher (double quencher)
Pan PV	any poliovirus
CV	coefficient of variation
